# Recent Advances on Immune Targeted Therapy of Colorectal Cancer Using bi-Specific Antibodies and Therapeutic Vaccines

**DOI:** 10.1186/s12575-021-00147-7

**Published:** 2021-07-01

**Authors:** Ali Azadi, Alireza Golchini, Sina Delazar, Fatemeh Abarghooi Kahaki, Seyed Mohsen Dehnavi, Zahra Payandeh, Shirin Eyvazi

**Affiliations:** 1 Department of Medicine, De La Salle Health Sciences Institute, Dasmariñas, Philippines; 2 Cancer surgery Department; Shiraz Medical School, Shiraz University of medical Sciences, Shiraz, Iran; 3 Department of Radiology, Tehran University of Medical Sciences, Tehran, Iran; 4 Department of Biotechnology, School of Advanced Technologies in Medicine, Shahid Beheshti University of Medical Sciences, Tehran, Iran; 5 Faculty of Life Sciences and Biotechnology, Shahid Beheshti University, Tehran, Iran; 6 Immunology Research Center, Biomedicine Institute, Tabriz University of Medical Sciences, Tabriz, Iran; 7 Department of Biology, Tabriz Branch, Islamic Azad University, Tabriz, Iran; 8 Biotechnology Research Center, Tabriz Branch, Islamic Azad University, Tabriz, Iran

**Keywords:** Colorectal cancer, Cancer vaccines, Bi-specific antibody, mRNA vaccines, Nanobodies, MGD007

## Abstract

Colorectal cancer (CRC) is a universal heterogeneous disease that is characterized by genetic and epigenetic alterations. Immunotherapy using monoclonal antibodies (mAb) and cancer vaccines are substitute strategies for CRC treatment. When cancer immunotherapy is combined with chemotherapy, surgery, and radiotherapy, the CRC treatment would become excessively efficient. One of the compelling immunotherapy approaches to increase the efficiency of CRC therapy is the deployment of therapeutic mAbs, nanobodies, bi-specific antibodies and cancer vaccines, which improve clinical outcomes in patients. Also, among the possible therapeutic approaches for CRC patients, gene vaccines in combination with antibodies are recently introduced as a new perspective. Here, we aimed to present the current progress in CRC immunotherapy, especially using Bi-specific antibodies and dendritic cells mRNA vaccines. For this aim, all data were extracted from Google Scholar, PubMed, Scopus, and Elsevier, using keywords cancer vaccines; CRC immunotherapy and CRC mRNA vaccines. About 97 articles were selected and investigated completely based on the latest developments and novelties on bi-specific antibodies, mRNA vaccines, nanobodies, and MGD007.

## Introduction

**Fig. 1:**
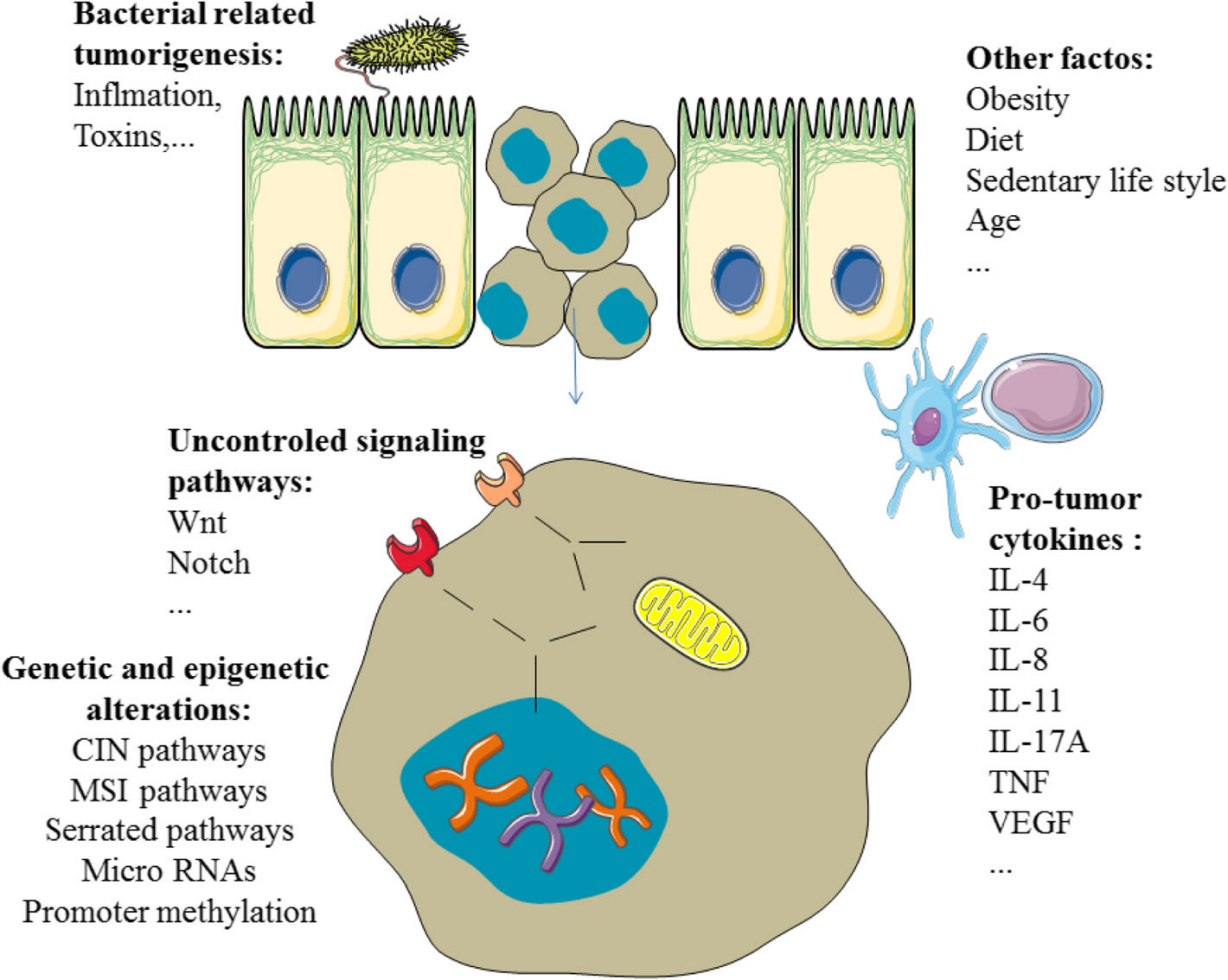
The different mechanisms associated with CRC promotion

Colorectal cancer (CRC) is a universal heterogeneous disease that is characterized by genetic and epigenetic alterations. The most reported genetic reasons for CRC are chromosomal and microsatellite instability (MSI), DNA hypomethylation, and mutation of oncogenes. The mutation rate of oncogenes can increase because of MSI. The chromosomal instability (CIN) pathway is the indication of chromosomal rearrangements and aneuploidy in cancers. The CIM pathway leads to the inactivation of tumor-suppressor genes [1–3]. The different mechanisms related to CRC induction are shown in Fig. 1.

The CRC patients are usually treated by neoadjuvant radiotherapy, adjuvant chemotherapy, and surgery. Surgery is not an appropriate treatment for more than 20% of patients with CRC; because of liver metastases at the same time of diagnosis. However, 5% of the patients die of recurrence or metastasis [4, 5]. Immunotherapy using monoclonal antibodies (mAb) and cancer vaccines are substitute strategies for CRC treatment [6]. When cancer immunotherapy is combined with chemotherapy, surgery, and radiotherapy, the CRC treatment would become excessively efficient [7, 8]. In CRC patients, stimulation of specific antitumor immunity can be accompanied by immune checkpoint blocking factors [9]. One of the compelling approaches to increase the efficiency of CRC therapy is the deployment of targeted nanoparticles as a drug delivery system which is under preclinical development. The aim of CRC immunotherapy is the induction of CD4+ and CD8+ CTLs in patients [10]. Dendritic cells (DCs) are powerful antigen-presenting cells (APC) that can be used in immune therapies to stimulate antitumor immune responses. Using immunotherapy for CRC patients is shown to be a promising approach but the main obstacles are the generation of the immune suppression synergistically and microenvironment. However, to maximize the antitumor immunity these strategies may be required to be combined with immune-modulating agents [11]. Activation of the T cells could be achieved by therapeutic strategies involving the blockade of immune checkpoints. Here, we aimed to present the current progress in CRC cancer immunotherapy, especially bi-specific antibodies and dendritic cells mRNA vaccines.

## Molecular Changes Associated with CRC

Multiple genetic mutations in germline and somatic cells lead to CRC. Different stages of the transition from normal tissue to cancer include the normal mucosa, through adenomatous polyp, to invasive cancer. Genome-wide analysis showed that several 100 genes have somatic mutations and a common of 80 mutations exist in any single CRC [12, 13]. Examples of gene mutations implicated in CRC are listed in Table 1.

**Table 1:** Some of the gene mutations implicated in CRC

Gene	Gene type	Consequence of mutation	References
APC	Tumor suppressor	loss of spindle microtubules regulation and increasing chromosomal instability	[14]
P53	Tumor suppressor	Loss of cell cycle regulation	[15]
RAS, BRAF PIK3CA	Oncogene	Increasing mutation in genes which involved in cell growth through MAPK pathway	[14–16]
WT1	Oncogene	significantly associated with tumor progression	[17]
MYH	Base excision repair	Cause somatic mutation of APC.	[18]

Tumor-associated antigens (TAAs) are displayed on the surface of cancer cells and significantly are over-expressed in the cells. Small peptides, which are derived from these TAAs, could bind to human leukocyte antigen (HLA). The bound peptides could be identified by T lymphocytes and initiate the anticancer response. Major TAAs in CRC are summarized in Table 2.

**Table 2:** Major TAA in colorectal cancer

TAA	Description	Reference
carcinoembryonic antigen (CEA)	Increased expression of CEA is associated with adenoma carcinoma mainly CRC	[19, 20]
Wilms’ tumor gene 1 (WT1)	Overexpression of WT1 gene plays a role in tumorigenesis of colorectal adenocarcinoma.	[17]
melanoma associated antigen (MAGE)	only expressed in cancer cells and testis including CRC MAGE-A4 induces an [21] immune response of CD4+ and CD8+	[22]
mucin 1 (MUC1)	Plays roles in self-renewal proliferation and self-renewal, drug-resistance, and anti-apoptosis and also invasion and metastasis of colorectal cancer stem cells	[23]
ring finger protein 43 (RNF43)	CTL-inducing peptide	[24–26]
outer mitochondrial membrane (TOMM34)	Combined chemotherapy of a RNF43 and TOMM34 peptide showed considerable result in a phase II study.	[27, 28]

Liu et al. investigated a mini-array of several TAAs, which was compounded of five TAAs containing Imp1, p62, Koc, p53, and c-myc full-length recombinant proteins. When CEA and these five anti-TAAs were handled together as biomarkers of colon cancer, the diagnostic sensibility elevates from 60.9 to 82.6% [29]. There are many molecular transformations in tumor cells. Identification of particular molecular transformations would benefit developing more efficient targeted therapies [30].

## The Application of Antibodies, Nanobodies and bi-Specific Antibodies in CRC Immunotherapy

A single B-cell clone is responsible for the generation of monoclonal antibodies (MAbs) which can bind to a single specific epitope. Köhler and Milstein set up a method for mAbs production called hybridoma method [31, 32]. MAbs have improved the clinical outcomes and patient survival, especially in inflammatory and neoplastic diseases [33].

**Fig. 2:**
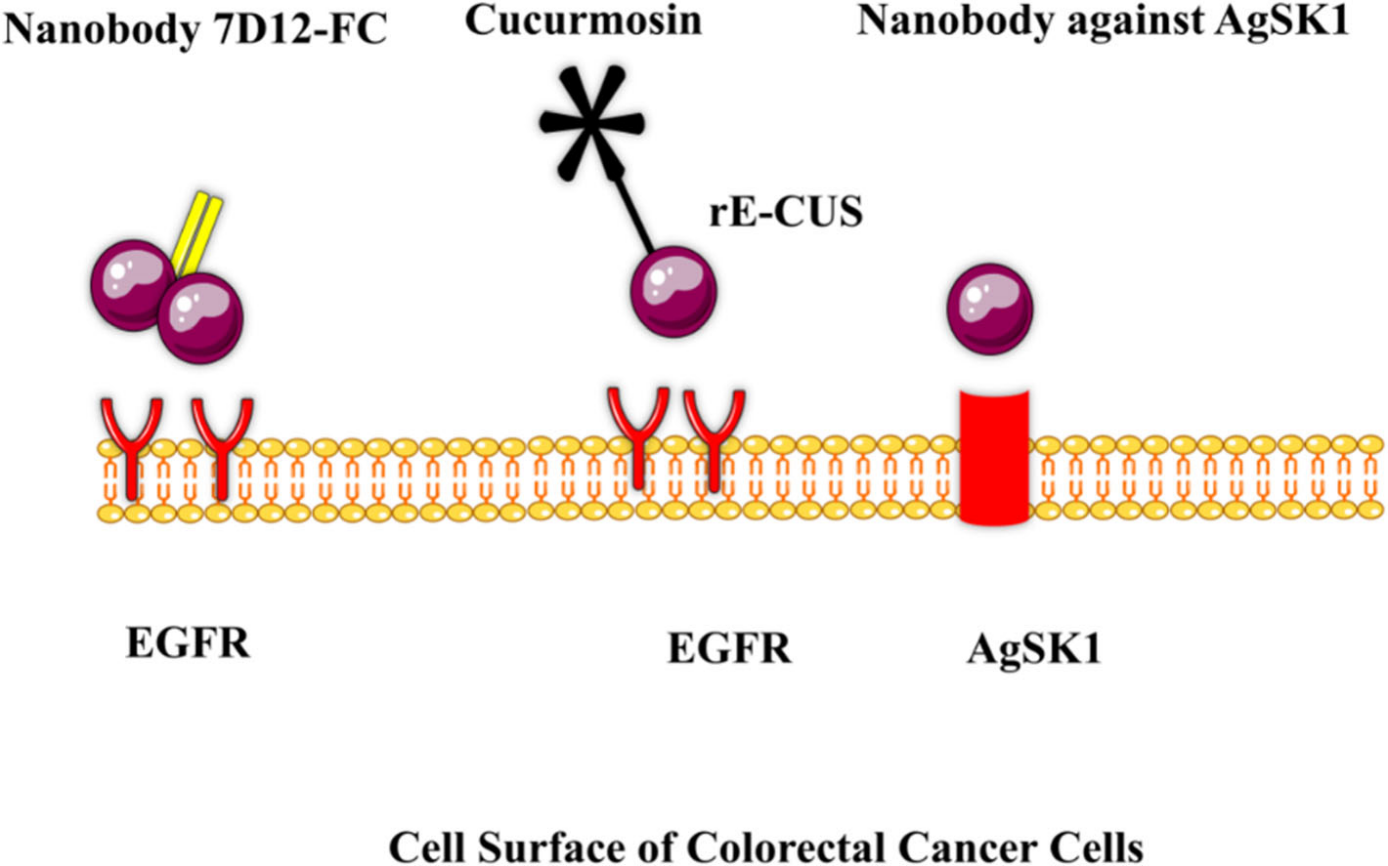
Different Nanobodies which have been used against CRC cells antigens

The mitogen-activated protein kinase (MAPK) pathway could be activated due to impaired function of the epidermal growth factor receptor (EGFR). The activated MAPK is vitally involved in the evolution of metastatic CRC (mCRC) [34]. Cetuximab and panitumumab are therapeutic anti-EGFR monoclonal antibodies developed to block the MAPK pathway, which is important for anti-mCRC therapeutics [35]. However, mutations in downstream signaling proteins cause primary resistance to anti-EGFR mAbs therapies [36]. A potential resistance-preventive strategy was achieved by using mixtures, which target different epitopes [37]. In this regard, Tintelnot et al. provided evidence that nanobodies have desirable characteristics [37]. The nanobody 7D12 attaches to a small epitope on EGFR that is consisted of amino acids which are involved in EGF binding [38, 39]. They showed that 7D12 fused to a human Fc domain, is able to obstruct EGFR with all examination obtained resistance mutations [40]. Their study demonstrated that the 7D12-hcAb nanobody can be a resistance-preventive therapeutic to target the EGFR pathway. Deng et al. constructed a novel immunotoxin expressed in Bacteria. It was specifically lethal against tumor cell lines with high levels of EGFR expression and suitable for treating EGFR-positive solid tumors. Their results showed that rE/CUS could be a potential therapeutic plan in treating EGFR-positive solid tumors [41]. Rashidi et al. isolated a nanobody against AgSK1 as a colorectal tumorassociated antigen which specifically reacted against colorectal cells [42]. All the mentioned studies are summarized in Fig. 2.

To overcome the deficiency of mAbs therapy, mAbs-based bi-specific antibodies have been introduced. The advantage of bi-specific antibodies is that they can simultaneously bind to two different antigens. The bi-specific antibodies can target two different receptors on the same cell and thus induce change in cell signaling [43]. There are two major formats of bi-specific antibodies, IgG-like formats, and non-IgG-like formats. IgG-like bi-specific antibody formats like catumaxomab have long serum half-lives. Catumaxomab can simultaneously bind to CD3and EpCAM. IgG-like formats provide Fc mediated effector functions, such as ADCC, CDC, and ADCP [44]. Blinatumomab is a bi-specific antibody of the non-IgG-like format; its small size enhances the tissue penetration and reduces the nonspecific activation of innate immune cells. However, the lack of Fc region in non-IgG-like formats reduced their half-lives. Food and Drug Administration (FDA) accepted the catumaxomab for the treatment of malignant ascites in patients with EpCAM-positive cancers [45]. In December 2014, Blinatumomab was approved for B cell malignancy [46]. More than 60 bi-specific antibodies are concurrently in preclinical trials and 30 bi-specific antibodies in clinical trials. About two-thirds of bi-specific antibodies are focused on cancer treatment [47]. Although bi-specific antibodies are being developed for diseases such as autoimmune, infectious, hemophilia, and Alzheimer’s disease, we mainly focused on recent advances about therapeutic bi-specific antibodies in CRC.

### Bi-Specific T-Cell Engagers (BiTE)

**Fig. 3:**
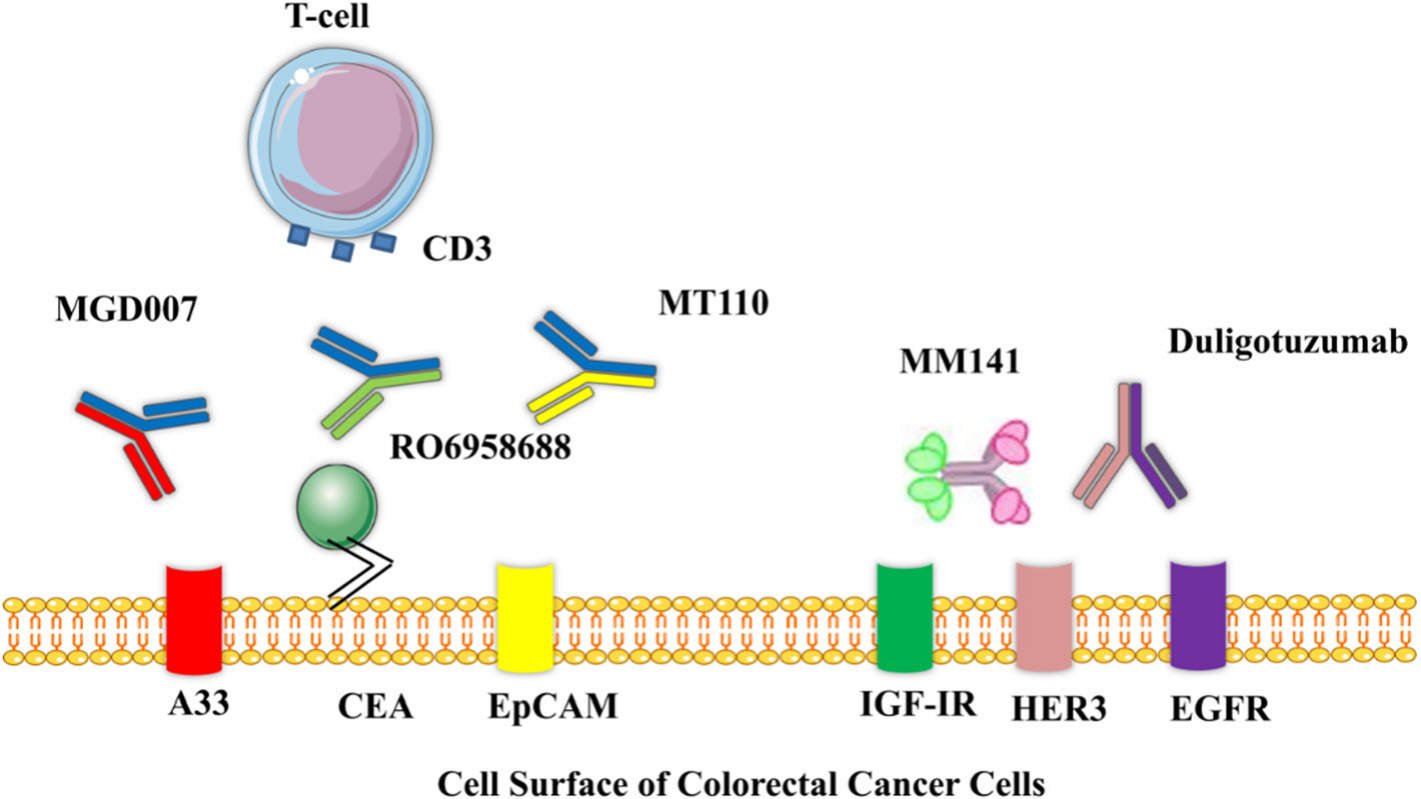
Different bi-specific antibodies which have been used against CRC antigens

BiTEs are single chain antibodies (scFv), produced by fusion of the minimal antigen-binding domains of two scFvs of different mAbs. One of the scFvs binds to T cells by the CD3 receptor and the other binds to a tumor cell by a tumor-specific molecule. BiTE directs a host’s immune system, especially T-cells, against cancer cells [48]. The small size of BiTE antibodies is optimal for the interaction between the T-cell and BiTE on the surface of the target cell. The following bi-specific antibodies are shown in Fig. 3.

### MGD007

MGD007 belongs to the DARTs protein (anti-glycoprotein A33/anti-CD3) and fusion of the Fc fragment to the DART molecule prolongs its serum retention time. A33 antigen is a glycoprotein that is expressed on the surface of more than 95% of human colon tumors and normal intestine. The glycoprotein A33 antigen is encoded by the GPA33 gene with homology to CAR and JAM as occluding junction-associated proteins. GpA33 is a potentially useful target for colon cancer [49]. The MGD007 is considered to co-engage gpA33-expressing CRC cells with CD3-expressing T cells and mediate powerful lysis of gpA33-positive cells. Currently, MGD007 is in phase I Clinical trial (NCT02248805) [50].

### RO6958688

RO6958688 Bi-specific antibody (anti-CEA/CD3) is produced with CrossMAb technology and is IgG1 based antibody. It has two Fab domains which capable of binding to CEA on tumor cells and CD3 on T cells, and the Fc region [51]. CEA is a glycoprotein that normally is produced during fetal development. The CEA is involved in cell adhesion and its production stops prior to birth. The CEA is normally present at very low levels in the blood of healthy adults and overexpressed in many tumors. RO6958688 induces cytotoxic T-lymphocyte reaction against CEA on tumor cells. The RO6958688 is on Phase I clinical study (NCT02324257) [52].

### MM-141 (Istiratumab)

MM-141 is an IgG-like Bi-specific antibody that has scFvs linked to the Fc fragment of an IgG. The MM-141 binds to the human epidermal growth factor receptor 3 (HER3) and insulin-like growth factor I receptor (IGF-IR). The HER family members are tyrosine kinases and are expressed widely in numerous cells. The HER family members activate the intracellular signaling pathways and consist of HER1, HER2, HER3, and HER4. Several cancers are associated with HER3 overexpression [53].

### Duligotuzumab (MEHD7945A/RG7597)

Duligotuzumab is an antibody that blocks ligand binding to EGFR and HER3 [54]. Duligotuzumab is demonstrated to induce ADCC using in vitro models and able to bind to Fc receptors [55]. HER1overexpression has been associated with advanced colorectal tumorigenesis [56]. The Duligotuzumab is able to bind to EGFR/HER receptors which can block the ligand-driven signaling from EGFR/HER2, EGFR/HER3, EGFR/EGFR, and HER2/HER3 dimer pairs [57]. Currently, phase II clinical analysis of the Duligotuzumab in together FOLFIRI (5-fluorouracil and irinotecan) is underway. This study showed improved overall survival or free survival compared to Cetuximab plus FOLFIRI in the second-line setting in patients with KRAS exon 2 wild-type metastatic CRC [58].

### MT110

MT110 is a specific antibody for EpCAM and CD3 which has been shown anti-tumor activity in animal models. Total elimination of TIC by MT110 has been demonstrated in vivo and in vitro by xenograft mouse model and a very sentient test by using measurement growth colonies on soft agar respectively. The EpCAM is overexpressed on primary and metastases of chiefly human adenocarcinoma [59]. The MT110 has potential ability to intercede absolute redirected lysis of colorectal TICs. The MT110 has shown an extremely effective eradication of pancreatic and colorectal tumor-initiating cells in preclinical trials [60]. MT110 is in phase I clinical trials [61].

### LY3164530

LY3164530 is an engineered anti-EGFR /c-MET Bi-specific antibody which inhibits the signaling by both EGFR and MET receptors. LY3164530 contains two identical light and heavy chains. Phase I study of LY3164530, in patients with advanced or metastatic cancer showed partial response in three patients with CRC. 17.2% of patients had constant disease≥ 4 months, the general response rate was 10.3%, and disease control rate was 51.7% [62].

## Immune Checkpoint Receptor

Immune response initiate with immune checkpoint receptors which prevent autoimmunity. Immune checkpoint receptors help T-cells as co-inhibitory and co-stimulatory to release cytokines [63]. Co-inhibitory immune checkpoint activates against tumor antigens to reduce the immune response in cancer cells. To date, different blocking agents have been designed to target CTLA-4, PD-1, and PD-L1.

### T-Lymphocyte-Associated Protein 4 (CTLA-4)

Regulatory T Cells (Tregs) expressed CTLA-4 (CD152) which prevents T cell functions. The CTLA-4 is upregulated on activated CD4^+^ T cells [64]. Antigen-presenting cells (APCs) express CD86 (B7–2) and CD80 (B7–1) which bind to CTLA-4and the CD28 molecules. The interaction between CTLA-4 and its ligands induces the trans-endocytosis of the CD80 and CD86 from the surface of APCs. Compared to CD28, the interaction of CTLA-4 is of higher affinity [65]. Unlike the CD28, CTLA-4 increases T cell activation and reduces the immune response against cancer cells.

Tremelimumab (CP-675,206) is a completely humanized IgG2which inhibits the T-cell activation by blocking CTLA-4 binding to B7.1 and B7.2 [66].. The Tremelimumab could be used as monotherapy or with other anticancer therapies and cancer vaccines [66]. The early clinical trials showed that the Tremelimumab induces durable objective tumor regressions.

### Programmed Cell Death-1 (PD-1)

The PD-1 immune checkpoint is expressed on T and B lymphocytes and bears homology to CTLA-4 [67]. PD-L1 and PD-L2 are PD1 ligands. The PD-L1 is present on hematopoietic and non-hematopoietic cells while PD-L2 is present on dendritic cells, macrophages, and certain B-cells [68–70]. PD-L1 negatively regulates the immune responses [71]. Current studies confirmed that the PD-1/PD-L1 is a target for tumor treatment strategizes [72]. Pembrolizumab and Nivolumab are PD-1 inhibitors that are accepted by the FDA [73]. Pembrolizumab is an IgG4 that attaches to the PD-1 and blocks its interplaying by PD-L1 and PD-L2. Nivolumab is derived from IgG4 and binds to PD-1 by affinity. Nivolumab can kill the activated T cells by activating the ADCC [74, 75]. Pembrolizumab was investigated in a phase I analysis. The results indicated that the patients were improved [76]. Phase I study demonstrated that Nivolumab has a low toxicity antitumor effect.

### Therapy with Combination of Anti-CTLA4 and Anti-PD1

The combination of CTLA-4 and PD-1 inhibitors could be a good approach for cancer therapy. CTLA-4 is activated earlier than PD-1, since for rapid responses, using anti-PD1 and anti-CTLA-4 antibodies respectively could lead to improved therapeutic activity. This approach was used by Weber et al.to treat melanoma and the results were promising. This combination therapy is suggested to be used in CRC treatment [77–79]. These agents showed promising results in clinical trials. Different Combination therapies have been assessed among tyrosine kinase inhibitors, chemotherapies, MEDI4736 (NCT02027961), MPDL3280A (NCT01633970, NCT02525757, NCT02409355) Nivolumab (NCT02464657, NCT01658878), and Pembrolizumab (NCT02551432) as targeted therapies [80]. In an attempt to convert the PD-1 checkpoint molecule to a T cell co-stimulatory receptor, Tang et al. have replaced the transmembrane and cytoplasmic tail of the PD-1 molecule with CD28 and 4-1BB signaling domains [81]. The combination therapies were also evaluated by targeting the oncogenic signaling pathways. The activated EGFR pathway inhibits the therapeutic effect of PD-1 blockade. It also is correlated with the upregulation of pro-tumor inflammatory cytokines, CTLA-4, PD-1, and PD-L1 [82].

Targeting a combination of inhibitory molecules on T cells has been recently assayed on a set of other checkpoint molecules, including CTLA-4, TIM-3, LAG-3, and T Cell immuno-receptor. This approach has led to the elimination of Tregs enhancement of effector function for T cells and elimination of MDSCs in the TME [83].

## Generation Mechanism of Antibodies, Nanobodies and bi-Specific Antibodies

### Generation Mechanism of Antibodies

Today, recombinant DNA technology is used for mAbs production. Transient or stable transfections are used to produce mAbs in mammalian cells. Transient transfections allow the rapid production of small quantities of product for use in the early stages of drug development. However, in large-scale industrial processing, stably transfected cell lines which are derived from a single cell clone are more commonly used. To produce high quantities of consistent products, manufacturing cell lines are transfected by plasmid vectors [84, 85]. The plasmid vector is available in a variety of designs, all of which are designed for mAb processing. Mammalian cells are the key workhorse for making the best and most important mAb products. Several methods can all be used to deliver plasmids. Positive transfectants are chosen for their drug resistance after transfection. Finally single clones are selected for scale-up and long-term expression characterization [86, 87].

### Generation Mechanism of Nanobodies

The performance of nanobodies is based on their robust,size and architecture, which is combined with the characteristic variance in the length of CDR3 [88, 89]. Nanobodies are typically generated by naïve, immune, and synthetic libraries. Because of the maturation of antibodies, immunization causes a wide range of high affinities [89], however the poorly controllable nature of immunization can obstruct selections against some protein.

In naïve and synthetic methods immunization stage was removed. Synthetic antibody libraries have a higher degree of control so they can be a better alternative for immune libraries. The advantages of naïve and synthetic methods immunization are: use for nonimunogenic targets, serves for different targets and the affinity and stability is improved. Phage, ribosomal and bacterial display methods are used to select antibodies with high affinity [88].

### Generation Mechanism of BsAbs

The latest design techniques for the development of BsAbs include quadroma technology, knobs-into-holes technology, CrossMAb technology, and protein engineering [90]. In Quadroma technology, two different hybridoma cell lines fuse to produce BsAb. Each of the hybridoma cells expresses its own specificity of mouse monoclonal antibody so the resulting BsAbs have two different arms with two specificities. BsAbs that produce from Quadroma have a long half-life, solubility and stability compare to normal antibodies. A disadvantage of this method is low efficiency due to producing nonfunctional antibodies. A chimeric quadroma cell line was created by combining rat hybridoma and murine hybridoma cell lines to solve the efficiency problem and produce antibodies that contain rat IgG2b and mouse IgG2a. Catumaxomab is the first BsAb that generate and approved with this method [91, 92].

In Knobs-into-holes (KiH) technology, the CH3 domain of an antibody is engineered to improve Fc heterodimerization. When engineering antibodies with this method some criteria such as distances between alpha-carbons, desirable conformationally and type of amino acid residues should be considered. Antibodies from this method have high stability, correct heterodimeric and can purify by protein A column [93, 94].

Bispecific antibodies, such as bi-, tri-, and tetravalent antibodies, as well as other novel Fab-based antibody derivatives, also can be produced using CrossMAb technology. The BsAbs consist of one modified unmodified arm. Modifications can be three formats. The first format includes replacing a heavy chain’s hole of Fab-arm with a cognate light chain from one half of a bispecific antibody. The second format includes replacing the VH of a Fab domain with its matching VL domain. Similarly, in the third format, the CH1 and CL of one arm of the bispecific antibody are swapped for proper heavy and light chain assembly [51, 95].

## Targeted Nanoparticles for CRC

Chemotherapy is reported to be highly effective in CRC treatment. However, chemotherapy is associated with various side effects such as hair loss. Therefore, it is now recommended to use these therapeutic agents in combination with monoclonal antibodies [96, 97]. Despite its promising nature, this combination therapy also suffers from challenges such as drug resistance [98]. Contemporary, researchers use nanoparticles as carriers of their pharmaceutical drugs [99]. These nanoparticles would decrease the side effects of cytotoxic drugs and improve their efficacy, solubility, pharmacokinetics, and bio-distribution. The first nano-carriers approved by US FDA are Liposome-based nano-products [100]. The liposome-based drugs for CRC treatment include CPX-1, LE-SN38 and ThermoDox [100]. Several agents are under preclinical development and have shown promising in vitro results for CRC treatment [101]. Conjugation of antibodies or fragments of antibodies on the surface of nanoparticles could make them more specific for cancer therapy. The main restraint for the inclusion of mAbs is their large size and complexity. The humanized A33 mAb (huA33 mAb) has shown great promise in CRC treatment [102]. In CRC cells the PD-L1 is express at a high level. In a phase II trial, MEDI4736 or MPDL3280A (both anti-PD-L1 antibodies) in combination with Cetuximab were used in advanced CRC [103].

Emami et al. halved enveloped doxorubicin (DOX) against CRC cells [104]. The antitumor activity of these nanoparticles plus NIR irradiation was estimated in CT-26 cells. Their results indicated that the employed approach has considerable potential to treat localized CRC [105]. Sharma et al. described a nano system based on methotrexate-loaded guar gum nanoparticles functionalized with folic acid (MTXFA- GGNP). In this system, methotrexate releases at colonic pH and displays preferential in vivo uptake in colon tissue [106]. Tissue-specific drug deliveries via PNPs avoid severe side effects against normal tissues and organs [107]. Gold nanoparticles have low cytotoxicity, biocompatibility, tunable surface features, easy functionalization, easy synthetization, and stability under most in vivo conditions. The nanoparticle surface can be modified with a wide range of functionalities which allows them to have specific drug targeting and controlled release [108]. Safwat et al. modified the gold nanoparticle surface with two thiol-containing ligands (thioglycolic acid (TGA) and glutathione (GSH)) to facilitate the 5-FU loading [109]. In 2019 Hao et al. used Branched Au-Ag nanoparticles with a polydopamine coating (Au-Ag@PDA) against a CRC cell line (HCT-116) and nude mice xenografts. These nanoparticles have shown strong near-infrared absorbance high photothermal conversion efficiency and no cytotoxicity. The obtained results suggested that this nanoparticle can inhibit cell proliferation and induced apoptosis in CRC cells via caspase-dependent and -independent apoptosis [110].

## Therapeutic Gene Vaccines for CRC

Various studies are currently in progress to develop vaccines for CRC treatment or prevention of recurrence after treatment. Different types of vaccines such as peptide [111], dendritic cells, DNA, RNA especially non-coding RNA and viral vaccine vectors are being basically investigated in phase I and II clinical trials [112]. Vaccination with protein moieties mainly contributes to humoral immune response, while confronting with cancer needs both cellular and the humoral immune response. Given these circumstances, DNA vaccines are being considered in tumor immunotherapy due to their ability to stimulate the production of CD8 T-cells. Unlike vaccines for infectious diseases, these vaccines are designed to augment the individual immune response, detect antigens, and confront cancer cells more effectively. The selection of appropriate tumor antigen is one of the most important steps in vaccine development. Various tumor antigens including CEA, MUC1, Sialyl-Tn, and SART3 antigens (which have higher expression in colon and rectum adenocarcinoma) are being investigated in clinical trials to vaccinate patients with CRC [113, 114].

Viruses such as the Vaccinia virus, poxvirus, and adenovirus directly infect and activate the APC cells. Therefore, they are deemed as suitable vectors to transfect tumor antigens. The results attained from clinical trials demonstrated the ability of these vectors to stimulate the cellular immune response against CEA, EpCAM/KSA, p53, and 5 T4 antigens related to CRC [112].

In a clinical trial conducted by Horig et al. patients with metastatic cancers were vaccinated with ALVAC-CEA-B7.1. In this vaccine, a non-replicating canary poxvirus (ALVAC) was engineered to express both the B7.1 co-stimulatory molecule and the CEA. This virus can infect human cells but it is unable to replicate. Designed ALVAC-CEA-B7.1 vaccine was injected intramuscularly every 4 weeks for 3 months and no toxicity and autoimmune response was detected. Findings indicated that the level of CEA-specific T cells was increased in 4 of 16 patients. Furthermore, antibody titer against ALVAC was assessed with a blood sample and ELISA method. The obtained results indicated increased antibody level after vaccination. The highest antibody level was observed in patients receiving the maximum dose of the vaccine [115].

TroVax or MVA-5 T4 vaccine was also used in phase II clinical trial. In order to design the vaccine, the gene encoding for the 5 T4 tumor antigen was inserted into the Modified Vaccinia Ankara (MVA) vector from the vaccinia virus. Previous studies revealed the ability of the resulted vaccine to induce immune response against antigens. The results of this trial showed that the injected TroVax vaccine was safe with no significant side effects. Antibody-specific response against 5 T4 and increased γ Interferon (IFN- γ) was also observed in 10 mCRC patients [116].

Using nucleic acid vaccines based on DNA or RNA [117] especially non-coding RNA [118] is a new strategy in immunogenicity which is under development. Various studies demonstrated the ability of nucleic acid vaccines in the induction of immune response against several diseases. Despite abundant information obtained from in vitro and in vivo studies, the efficacy of DNA and RNA vaccines in CRC has not been thoroughly studied in clinical trials [119].

In a study on DNA vaccine in phase I clinical trial, CEA tumor antigen-encoding plasmid (pCEA/HBsAg) and Hepatitis B Surface Antigen (HBsAg) were used in 17 patients with m CRC. Repeated doses of DNA vaccines induced HBsAg antibody production in 6 of 8 patients and increased the levels of protective antibodies in 4 patients. Although 4 of 17 patients presented Lympho-proliferative response against the CEA, the CEA-specific antibody response was not observed after vaccination. In this study, clinical responses against the vaccine were not observed among 17 patients with m CRC [120].

In a phase I study, Staff et al. used altered CEA antigen-encoding plasmid DNA vaccine (CEA66 DNA) in combination with T helper cells-related epitope. The most reported level 1 and 2 complications included reaction at the injection site, fatigue, headache, joint pain, chest cramps, and muscular pain. As a result, the CEA66 DNA vaccine was tolerated well and there was no sign of autoimmune response [121].

In a phase I and II clinical trials on RNA vaccines, the safety and feasibility of vaccination with autologous dendritic cells transfected with CEA tumor antigen-encoding mRNA were investigated among CRC patients with metastatic liver surgery. In this study, CEA mRNA-transfected DCs (DC-CEA) were used. Results revealed that the immunogenicity was tolerated well and one out of 24 assessed patients (in phase I) had a complete response, two patients were with limited response, 3 patients had stable disease, and 18 patients had progressive disease. In Phase II of the study, 9 out of 13 patients experienced a recurrence of the disease in 122 days. Evidences demonstrated the induction of immune response in samples obtained from the dendritic cell injection site and peripheral blood. According to the obtained results, it can be said that loaded mRNA in dendritic cells is safe and feasible in patients with m CRC [122, 123]. Therapeutic gene vaccines for CRC summarized in Table 3.

**Table 3:** Therapeutic gene vaccines for CRC

Vaccine	DNA or mRNA	Mechanism of action	company	Result	Reference
MYB	DNA	The vaccine contains a gene cassette of a hybrid with tetanus toxin gene sided an MYB gene which was inactivated.	Peter MacCallum Cancer Centre	Safe, MYB transcription factor is highly pressed in epithelial cancers cell	[124]
pcDNA-hNIS	DNA	IgG antibody titers were significantly increased	under investigation in clinical trials	displayed encouraging antitumor effects	[125]
pVAX1-HER2	DNA	in 11.4% of colorectal adenocarcinomas, HER2 protein expressed	under investigation in clinical trials	colorectal adenocarcinomas Patients found better condition	[126]
CpVR-MS and CpDV-IL2-MS	DNA	CCL-19 and GM-CSF upregulated. - A CpG and interleukin-2 (IL-2) was used as adjuvants.	under investigation in clinical trials	In mice 69.1% of colorectal carcinoma-bearing was suppressed	[127]
pCEA/HBsAg	DNA	Promoting a promising immune system	under investigation in clinical trials	No detectable CEA- antibodies was seen in patients	[120]
CEA66 DNA	DNA	Stimulation of Cellular and humeral response against CEA	under investigation in clinical trials	75% of showed detectable CEA immune responses.	[128]
NCI 4650 (mRNA 4650)	RNA	Immunostimulants	Moderna Therapeutics	safe and induce CD8 and CD4 T cell responses	[129]
RO7198457 (RG 6180)	RNA	Immunostimulants	BioNTech	Safe and neoantigen-specific immune responses	[130]
mRNA 4157	RNA	m-RNA 4157 Indoctrination for peptides containing exclusive mutations (i.e., neoantigen) present in each patient’s specific tumor.	Moderna’s /Merck	Safe, well tolerated and T cell responses	[131]
DC-CEA	RNA	tumor antigen-encoding mRNA was transfected to autologous dendritic cells	Moderna	immunogenicity was tolerated suitable	[132]
V 941 (mRNA 5671)	RNA	This vaccine contains epitopes for KRAS mutations (G12D, G12V, G13D, and G12C T cell immune responses.	Moderna	The findings are keenly predictable.	[133]

## Vaccine and Antibody Combination Therapy for CRC

CRC is highly prevalent all over the world [134]. In some cases, colon cancer metastasizes to the other organs’ tissues such as the liver, lung, and peritoneum [135]. Therefore, overcoming this type of cancer is highly important [134]. Besides, using vaccines along with mAbs could efficiently fight tumors. In a study by Hoffmann et al. (2007), fusogenic membrane glycoproteins H and F (MV-FMG) encoded by HSV-1 vector were used lonely or together with Cetuximab. The observations showed that expression of MV-FMG induces cell-cell fusion and synergistically enhanced the cytotoxicity of Cetuximab [136]. In another study by Wu et al. (2016), a TM4SF5-specific peptide vaccine and the anti-TM4SF5 monoclonal antibody were used to inhibit the metastatic potential of colon cancer in a mouse model. It was shown that immunization with the TM4SF5-specific peptide vaccine reduces the growth of lung tumors and improves the survival rate. In addition, the humanized mAb was reactive to the cyclic peptide. This mAb showed effective in vitro anti-cancer properties. However, the use of peptide vaccines in combination with TM4SF5 mAb significantly reduced metastatic colon cancer in mice [137].

Although it is expected that the use of vaccines in combination with mAbs could enhance the efficacy of therapy, only a few studies evaluated this combination therapy. Therefore, it could be suggested that studies should expand over this strategy not only in CRCs but also in a broad range of other human cancers.

## Conclusion

CRC is a common malignancy in the world. Immunotherapy using mAb and cancer vaccines are putative strategies for CRC treatment. Combined CRC therapy using chemotherapy, surgery, and radiotherapy enhances the efficacy of CRC treatment. As discussed above therapeutic mAbs, nanobodies and bi-specific antibodies promote the efficiency of CRC treatment strategies.

Moreover, therapeutic cancer vaccines have been demonstrated to improve the clinical outcomes of patients. Therefore, it could be concluded that combining the therapeutic efficacy of monoclonal antibodies and gene vaccines could be deemed as a novel perspective in CRC treatment.

## Data Availability

All data and materials are within the paper.

## Abbreviations

CRC: Colorectal cancer
MSI: Microsatellite instability
mAb: monoclonal antibodies
DCs: Dendritic cells
APC: Antigen-presenting cells
TAAs: Tumor-associated antigens
HLA: Human leukocyte antigen (HLA)
mCRC: metastatic CRC
EGFR: Epidermal growth factor receptor
MAPK: Mitogen-activated protein kinase
FDA: Food and Drug Administration
BiTE: Bi-specific T-cell engagers
